# Underestimating College Student Food Insecurity: Marginally Food Secure Students May Not Be Food Secure

**DOI:** 10.3390/nu14153142

**Published:** 2022-07-29

**Authors:** Stephanie A. Brescia, Cara L. Cuite

**Affiliations:** 1 Graduate School of Education, Rutgers University, New Brunswick, NJ 08854, USA; 2 Department of Human Ecology, Rutgers University, New Brunswick, NJ 08854, USA; cuite@sebs.rutgers.edu

**Keywords:** marginal food security, college student food insecurity

## Abstract

The purpose of this study was to understand the demographic, student, financial, and academic differences between marginally food secure college students and students with high, low, or very low food security (FS). Unlike highly food secure students, marginally food secure students worry about the quantity and quality of their food, yet they are classified in the same category as highly food secure individuals as per the United States Department of Agriculture (USDA) reporting standards. To investigate marginal FS among college students, a cross-sectional online survey was administered at a large, public, research university in the Northeastern United States. A largely representative sample of 6823 undergraduate students completed the survey with a 19.7% response rate. Self-reported level of FS was measured using the validated USDA 10-item FS survey module. Independent variables, such as demographic and student characteristics and cumulative grade point average (GPA), were gathered from institutional databases, and self-reported mechanisms of financing education were measured using a novel scale. Results from the multinomial logistic regression revealed statistically significant differences in GPA between students with marginal and high FS (*p* < 0.001), but not between students with marginal and low FS (*p* = 0.31). This work has implications beyond college students and suggests that marginally food secure populations should not be labeled as food secure.

## 1. Introduction

Sometimes described as the hidden subcategory between food security (FS) and insecurity (FI) [1], marginal FS is underexplored in the general and college student populations. Unlike individuals who are highly food secure and do not experience any indications of food insufficiency, those who are marginally food secure tend to worry about the quantity and adequacy of their food supply [2]. There are noted differences between people with high and marginal FS [2,3,4], yet they are often considered to be the same as per the United States Department of Agriculture (USDA) reporting standards. Households with high or marginal FS are labeled “food secure”, while those with low or very low FS are “food insecure”, meaning they lack consistent access to enough food for healthy living [5]. 

Most of the literature focuses on people who are members of the latter two categories, experiencing low or very low FS, with little insight into the potential consequences of marginal FS. If marginally food secure students are different from highly food secure students in terms of their ability to acquire enough food for healthy living, a trend that has been documented in the general population [6], it is possible that scholars are underestimating the proportion of college students who may need food support or other resources [7]. The purpose of this study is to address the gap in the extant literature by examining the demographic, student (i.e., full-time or part-time enrollment and first generation college student status), financial, and academic characteristics that vary between students with marginal FS and students with high, low, and very low FS. Ultimately, the goal is to shed light on whether it is helpful to group students with marginal FS with other food secure students or if they should be considered more similar to food insecure students. 

### 1.1. Measuring Marginal FS

The USDA 10-item FS questionnaire is commonly used to measure FS [8]. Each affirmative response constitutes a score of 1 point, and depending on the total number of affirmative responses given, individuals are placed into one of four categories: high, marginal, low, or very low FS. As described in Table 1, highly food secure individuals have a total score of 0 and do not have any indication of FI. Marginally food secure individuals have scores of 1 or 2 [9], which typically represent anxiety over the sufficiency of one’s food supply (USDA, 2022). Members of this group tend to affirm one or both questions, “I worried whether my food would run out before I got money to buy more” and “The food that I bought just didn’t last and I didn’t have money to get more”. Unlike individuals with low (scores of 3–5) or very low (scores of 6–10) FS, those experiencing marginal FS typically do not report reduced diet quality, variety, or food intake.

In 2020, 89.5% of the US population was food secure [5], but among the households deemed food secure, 9.7% of households with children and 6.2% of households without children reported one or two indicators of FI, such as worrying about their food supply or not having enough money to buy more food, meaning their FS is classified as marginal [10]. The USDA reported that 14.2% of all households affirmed that they worried their food would run out before having money to buy more and 11.4% reported that the food they bought did not last, both indications of at least marginal FS, if not more [10].

According to USDA classifications [10], individuals can affirm some level of FI while still being considered food secure. The decision not to include less severe indicators, such as worry over food supply, under the label food insecure has serious implications. Hamilton and colleagues [11] explain,

This strategy reduces the likelihood that a household will be placed in a too severe category of food insecurity because of an erroneous affirmative response (a “false positive” classification). The trade-off is an increased likelihood that a household will be placed into a less severe category than actually merited (a “false negative” identification) (p. 38).

Based on this process, it is likely that FI is underreported in the United States [3], which has important implications for members of the general and college student populations.

### 1.2. Implications of Marginal FS in the General Population

Marginal FS is understudied in the general population, but the limited research in this area provides evidence that marginal FS, unlike high food FS, is associated with negative health outcomes. Analyzing data from the National Health Interview Survey, researchers found that low and very low FS was significantly associated with 10 chronic diseases including hypertension, cancer, stroke, diabetes, and kidney disease among other life-threatening ailments [12]. Adults from marginally food secure households were 5.8 percentage points more likely to have a chronic disease than adults from highly food secure households, and compared to their highly food secure counterparts, marginally food secure adults were 9.0 percentage points less likely to report excellent health [12]. Other studies have also found differences in mental health concerns, such as anxiety and depression, by FS level. Mothers with marginal FS are three times more likely to report depressive symptoms compared to mothers with high FS [13]. Another study [14] found that when adjusting for selected demographic covariates, 21.0% of marginally food secure mothers experienced anxiety or depression, while 16.9% of highly food secure mothers and 36.7% of food insecure mothers reported the same conditions. Collectively, these findings suggest that marginal FS results in worse physical and mental health than high FS.

Differences between marginally food secure homes and highly food secure homes also impact academic outcomes in children. In a study that utilized a modified version of the USDA’s FS index, where marginally food secure households were considered food insecure, investigators found that FI during kindergarten was related to poor academic performance [15]. Another study that also focused on kindergarten children found that FI has a negative effect on fall math scores and overall learning [16]. These effects are similar among marginally food secure homes and food insecure homes. The authors conclude, “children begin to experience the effects of food insecurity even at the most marginal level of household food deprivation” (p. 145) [16]. Members of marginally food secure households experience negative health and personal outcomes, leading many to conclude that it is inappropriate to cluster marginally food secure households in the same category as highly food secure homes [3,4,13]. Results from a large, national study of FI demonstrate that marginally food secure households are statistically different from highly food secure households, especially in regard to household income, the level of education of the head of the household, and average weekly food expenditures [3]. These substantial differences suggest that marginally food secure households have unmet food and financial needs, which is problematic because estimates of FS are commonly used to allocate resources for public support programs. Coleman-Jensen [3] concludes,

…if marginally secure households are experiencing food anxiety at the time of data collection they are likely to soon experience more severe conditions of FI such as reduced food intake. Even if current situations of marginally secure and insecure households are different, their long run outcomes are likely to be similar and they are likely to display similar needs in terms of food and economic assistance (p. 228).

Recommendations from this research include using separate categorizations for marginally and highly food secure households for a more nuanced understanding of the outcomes and conditions associated with marginal FS [3].

### 1.3. FI in the College Student Population

More than one third of college students are food insecure nationwide [17]. Among students with low or very low FS, there is an overrepresentation of racial and ethnic minorities, former foster youth, and first-generation college students [17]. Prior research demonstrates that three-quarters of food insecure students receive financial aid, with more than half receiving the Pell Grant [18], a form of federal financial aid reserved for the most financially under-resourced students [19]. Students who do not have financial support from their families or are financially independent are more likely to experience FI compared to students with familial financial assistance [20].

Research also suggests that food insecure students earn lower grade point averages (GPAs) than those who do not experience food difficulties [21]. One study found significant differences in GPA, with food insecure students having a 3.33 average and food secure students having a 3.51 average out of 4.0 [22]. Another study explored the intersectional identities of students and determined that Black and Latinx students are more likely to experience FI and may have lower GPAs, due, in part, to their FI status [21]. Other studies have linked FI to difficulty studying [23] and a decreased likelihood of college completion [24].

While the body of research surrounding college student FI is growing, there is little insight into the potential consequences of marginal FS among college students. This is especially problematic given the negative outcomes that have been associated with marginal FS in the general population. To our knowledge, there is one peer-reviewed publication that has explored marginal FS in the college student population [7]. Investigators found that marginally food secure students were statistically different from highly food secure students in terms of age, race/ethnicity, employment status, and perceived health, among other characteristics [7]. If investigators had used a binary FS variable (i.e., food secure or insecure), the differences between highly and marginally food secure students would be lost. While the work of Soldavini and Berner [7] provides important contributions to the literature, further exploration of the differences between marginally food secure students and students with high, low, or very low FS, specifically in regard to their academic performance, is needed.

Differences between individuals with marginal and high FS have been documented in the general population, and this study will fill a gap in the extant literature by exploring marginal FS in the college student population. Determining if differences between groups also exist among students is an important contribution that could lead to a more nuanced understanding of marginal FS and the resources needed to support this population. Marginally food secure students are not free from food access challenges in the same way as highly food secure students, but they also do not experience the same depth of food insufficiency as those with low or very low FS, making them an intermediary between these groups. Statistically significant differences between students with marginal FS and students with all other levels of FS (i.e., high, low, and very low) were explored. The following research question guided this investigation:

Are marginally food secure students statistically different from students with high, low, or very low FS in terms of demographic and student characteristics, mechanisms of financing education, and cumulative GPA?

## 2. Materials and Methods

### 2.1. Procedure

This study was conducted at a large, public, 4-year research university (i.e., an institution that offers 4-year academic programs that lead to a bachelor’s degree) where 36.9% of undergraduates were food insecure in 2016 [25]. Findings from the cross-sectional study indicated that students who identified as Black, Hispanic, or other, had parents with lower levels of education, and were not US citizens were more likely to be food insecure. In addition, undergraduates who received a Pell Grant, were financially independent, and had a job other than a work–study position had a heightened chance of FI. Finally, researchers also found differences in GPA by level of FS [25].

Approval from the Institutional Review Board (IRB) was granted prior to conducting this study (Rutgers University New Brunswick Arts and Sciences IRB, Protocol IRB# e16-737, Approved 9 June 2016). A cross-sectional, online census survey was used to conduct this study because it allows for effective and efficient data collection about an issue that is critical to student success. Furthermore, it is the most common research methodology used to study student FI [26]. Selecting methodology that is aligned with methods used at other institutions and research centers that study student FI at the national level allows us to make national and interinstitutional comparisons.

The survey was administered in collaboration with the offices in charge of student affairs and institutional research, and a senior administrator sent an email invitation to participate in the web-based survey to all matriculated graduate and undergraduate students (43,779 in total) on 22 November 2016. The email explained that the university was interested in learning more about student experiences with hunger and FI. The survey took approximately 10 min to complete and students that finished were entered into a lottery to win one of four USD 100 gift cards. Subsequent follow-up emails encouraging participation were sent periodically.

Survey responses were collected through 19 December 2016. A total of 8393 undergraduate and graduate students completed the survey, yielding an overall response rate of 19.17%. Because of significant differences in the predictors of FI between undergraduate and graduate students [25], this investigation focuses on the undergraduate population only, which consisted of 6823 respondents for a 19.7% response rate.

### 2.2. Survey Instrument

Students logged into the survey platform using their student identification number. The survey instrument included a detailed consent form. Students were informed that if they did consent to participation, the investigators would receive information about them from the institutional database including year in school based on the number of credits completed, type of enrollment (part-time or full-time), cumulative GPA, age, sex (male or female), race/ethnicity (African American, Asian, Hispanic, White, and other comprised of Native American/American Indian, two or more, other, and unknown), and citizenship (U.S citizen or not). In addition, parents’ educational level (no high school, some high school, high school graduate, some college, 2-year degree, 4-year degree, and graduate study) was used to indicate first-generation status, defined as neither parent having a bachelor’s degree. An institutional research staff member matched survey data to institutional data using student identification number and provided investigators with de-identified data.

Since this was the first time student FI was investigated at the research site, the survey instrument consisted of both exploratory and validated measures. Questions that were exploratory were specific to the university where the investigation took place and pertained to student meal plans and cafeteria use, personal finance, place of residence, and one open-ended question. All exploratory questions relied on self-reported information. The instrument also contained the USDA’s validated 10-item FS scale to determine each respondent’s self-reported FS status: high (scores of 0), marginal (scores of 1–2), low (scores of 3–5), or very low (scores of 6–10) FS (see Table 1). Responses to these questions were scored in accordance with guidelines provided by the USDA [9], where higher numerical scores are associated with more severe FI.

### 2.3. Sample Representativeness

A staff member from institutional research conducted a series of chi-square tests of goodness of fit to determine whether the full undergraduate sample (*n* = 6823) was representative of the larger student body in regard to citizenship, class level, sex, degree level, school, enrollment status, race, and financial aid (see Table S1). There were no statistically significant differences for race. There were statistically significant differences for all other subgroups, and Cramer’s V statistics were calculated to determine the effect size of these differences. All subgroups, with the exception ofsex, had Cramer’s V statistics of less than 0.10, indicating a minor effect [27]. Based on these analyses, the sample is considered generally representative of the institution’s undergraduate student population, with minor differences in terms of sex.

### 2.4. Sample Description

A complete description of the sample is contained in Table 2. Only undergraduates that had a GPA (first-time freshmen and transfer students did not have a GPA in Fall 2016 and are not included in the sample) at the time of data collection and were not missing data for any of the independent variables under investigation (*n* = 4491) were included in analyses. Nearly half (42.2%) were highly food secure, 16.6% were marginally food secure, and 41.1% were food insecure (18.2% with low FS and 22.9% with very low FS). The sample consisted of mostly U.S. citizens enrolled full-time. Approximately one-third of students were the first in their families to attend college. The majority of students (73.2%) indicated that they had some financial support from family members to cover the cost of educational expenses, including tuition, fees, and living expenses. More than half (67.2%) reported that they finance their education with loans, savings, or credit cards, while fewer students, 39.8%, reported receiving a grant, scholarship, or fellowship. More than a quarter of students had need-based federal financial aid in the form of Federal work-study or a Pell Grant. A few students were connected to government food benefits or food pantries on or off campus. The average cumulative GPA (i.e., a student’s average GPA throughout all semesters of enrollment) of respondents was 3.18 (SD = 0.55) out of a maximum of 4.0.

### 2.5. Data Analysis

Statistical Package for the Social Sciences (SPSS) version 26 was used to conduct all statistical analyses [28]. Multinomial logistic regression was used to determine the effects of independent variables (demographic and student characteristics, mechanisms of financing education, and cumulative GPA) on students’ level of FS (high, marginal, low, or very low). This analysis is appropriate for a multicategorical ordinal outcome variable [29], and was used by the only other two papers to investigate marginal FS [3,7] to conduct similar analyses.

Marginal FS was the baseline reference category that was compared to high, low, and very low FS to identify statistically significant differences between each pair of groups (i.e., marginal vs. high FS, marginal vs. low FS, and marginal vs. very low FS). For each binary comparison, odds ratios (OR), standard error (SE), and 99% confidence intervals were reported. Prior to executing the multinomial model, collinearity diagnostics were conducted. The variance inflation factor (VIF) and tolerance statistic were used to determine if multicollinearity exists. VIF values greater than 10 [30] and tolerance values below 0.1 [31] suggest that there is a collinearity issue.

We used a Bonferroni correction to decrease the likelihood of Type I error given the multiple comparisons conducted [32]. Given the three sets of comparisons in the model, we divided the standard *p* < 0.05 by the number of comparisons to reduce the family-wise error rate. Thus, our alpha was set at *p* < 0.01 (0.05/3 = 0.017).

## 3. Results

### 3.1. Overall Model

A total of 4491 cases were included in the model. Collinearity diagnostics revealed that independent variables were not highly correlated with each other (VIFs all <1.42 and tolerance all <0.7). To determine the overall ability of the model to predict FS, a forced entry main effects model was created with all variables entered into the model at the same time. The coefficient of determination, Nagelkerke’s R^2^, was 0.158, meaning that 15.8% of the variance in the dependent variable (level of FS) can be explained by the model. As seen in Table 3, all independent variables, with the exception of age, (x^2^ (3) = 7.180, *p* < 0.066), and having a non-work-study job, (x^2^ (3) = 10.347, *p* < 0.016) were statistically significant. To more fully understand which variables are significant determinants of marginal FS, and to compare each pair of outcome categories, individual parameter estimates were interpreted. The results of the pairwise comparisons are contained in Table 4.

### 3.2. High FS vs. Marginal FS

While students with high and marginal FS are both considered food secure, there were five statistically significant differences across these groups, as illustrated in Table 4. Citizens were 1.6 times more likely to be highly food secure than non-citizens. In terms of how students cover the cost of their educational expenses, including tuition, fees, and living expenses, students who have financial support from their families were 1.3 times more likely to be highly food secure, while students that use loans, savings, or credit cards (using loans, savings, or credit cards is categorized under the “finance education” variable) were 1.4 times less likely to experience high FS. Students who had a work-study position or a Pell Grant, two types of need-based financial aid, were nearly 1.5 times less likely to be highly food secure. The GPAs of students with high and marginal FS were also statistically different (*p* = 0.001). For each 1 unit increase in GPA, students were 1.4 times more likely to be highly food secure.

### 3.3. Low FS vs. Marginal FS

Students with low and marginal FS are categorized differently as per the USDA, where marginally food secure individuals are food secure and individuals with low FS are food insecure. However, of all the groups, students with low and marginal FS are the most similar. There were only three statistically significant differences at the 0.01 level (see Table 4). Specifically, Hispanic students were roughly 1.5 times more likely to have low FS than White students. Having friends who help pay for educational expenses increased the likelihood of low FS by 2.3 times, while having a grant, scholarship, or fellowship may insulate or protect students from experiencing low FS with an odds ratio of 0.733 (1.4 times less likely to have low FS).

### 3.4. Very Low FS vs. Marginal FS

Table 4 indicates that there were six statistically significant differences between students with marginal vs. very low FS, the latter group typically reporting severe food acquisition difficulties. Notably, when compared to marginally food secure students, individuals were more likely to report very low FS if they were enrolled full-time rather than part-time (2.6 times greater likelihood) or were Hispanic (1.6 times greater likelihood). Financially, students are 1.4 times less likely to have very low FS if their family members assist them with educational expenses. Receiving assistance from friends or paying for school with loans, savings, or credit cards increased their odds of experiencing very low as compared to marginal FS by 4.9 times and 1.5 times, respectively. Statistically significant differences in GPA were expected and observed. As GPA increased by 1 unit, students became 1.5 times less likely to have very low FS, indicating that higher GPAs are associated with higher levels of FS.

## 4. Discussion

This study and others [3,7] suggest that individuals who are marginally food secure are likely to experience some of the detrimental effects that accompany FI even though they are typically classified as food secure. Marginally food secure students were more similar to low food secure students (i.e., fewer statistically significant differences were observed) than marginally food secure students were with highly food secure students with whom they typically share the food secure designation. These findings indicate that the common practice of grouping marginally food secure students with highly food secure students may not be appropriate. This leads to our main recommendation that marginally food secure individuals, at least within the college student population, should not be labelled as food secure. Pertinent findings and the implications for FI measurement among college students and the general population are discussed.

### 4.1. Investigating Student and Demographic Characteristics

Marginally food secure students were similar to highly food secure students in terms of demographics, with the exception of non-citizens having been more likely to experience marginal FS than citizens. This is consistent with the literature on non-citizen students being more likely to be food insecure [7,33,34,35]. However, this finding should be interpreted with caution due to the small percentage of students in the sample who were non-citizens (11.7%).

Institutions may want to investigate other student characteristics that have been linked to higher rates of FI, such as being a parenting student or former foster youth [33], to help them determine the number of students who may be in need of support on their campus. Having additional information about student characteristics that are associated with marginal FS will provide insight into who is more likely to experience the detrimental academic effects associated with these conditions.

### 4.2. Re-Envisioning Financial Aid

Colleges and universities cannot control how students and their families decide to pay for educational and living expenses. However, institutions can use existing research [36,37,38] that shows that FI is related to how students pay for college, along with insights from this study to understand the association between mechanisms of financing education and levels of FS. The only financial variable that increases a students’ likelihood of being highly food secure rather than marginally food secure is having financial support from family members. Financial aid administrators are unable to determine which students have parental support, but they can identify the students who are deemed financially independent as per their Free Application for Federal Student Aid (FAFSA). These students should be informed of existing campus or community resources that could help them secure additional food.

Other pertinent findings point to the potential inadequacy of the financial aid system. Just as other studies have shown that students who receive Pell Grants or Federal work-study are more likely to be food insecure [21], they were statistically more likely to be marginally food secure than highly food secure in the current study. Taken together, these findings dismantle the idea that need-based financial aid provides ample assistance to under-resourced students. Increased allocations to need-based aid programs are warranted.

### 4.3. Providing Food Supports to Improve GPA

FI is linked to poor academic performance among college students [21] and the current study suggests that marginally food secure students also experience negative effects from their FS status on academic outcomes. As demonstrated by statistically significant differences in GPA among students with high and marginal FS, even worrying about affording food is linked to lower GPAs. Furthermore, there were no statistically significant differences in GPA between students with low and marginal FS, meaning marginally food secure students were performing similarly to students who have demonstrated food difficulties.

Differences in GPA could be caused by a multitude of factors, some of which may be related to FS and some not. These can include inadequate pre-college preparation, personal or familial stressors, or working more than 20 hours per week. While level of FS is not the only factor influencing student academic performance, there is cause for concern. Numerous other studies have demonstrated an inverse relationship between FI and GPA [22,39], and this study extends these findings as GPA was lower for students who affirmed just one or two indicators of FI. This means that in addition to students with low or very low FS, marginally food secure students could potentially earn lower grades due, at least in part, to their level of FS. Research demonstrates that students with lower grades have an increased likelihood of leaving school [40], meaning they could potentially incur large amounts of debt without experiencing the financial benefits afforded by a college credential. This provides yet another reason for colleges and universities to provide programming such as food pantries and increased financial resources to address FI [35].

### 4.4. Limitations

While a strength of the study is that we were able to identify that the sample is representative of the undergraduate student population, there are limitations. Student participation in the survey was voluntary, and certain factors could have caused the number of food insecure students in the sample to be underrepresented. The survey was administered in late November, towards the end of the fall semester, meaning food insecure students may have already left the institution due to food or financial challenges. Furthermore, food insecure students often have many responsibilities including working and caring for children, which could limit the time they have to complete a survey [41]. However, other design elements such as the identification of the survey topic in the email invitation or the lottery incentive could have caused an overrepresentation of food insecure students.

Data regarding demographic and student characteristics and cumulative GPA were retrieved from student institutional records and included in the multinomial analysis. While the likelihood of inaccuracies in these data are minimal, all the mechanisms of financing education were based on student self-reported data and the scale used to collect this information was not validated. It is possible that students may have had difficulty identifying how their educational expenses were paid or that the scale did not appropriately capture the information it was designed to measure. Potential inaccuracies in self-reported financial data could influence the results from the multinomial logistic regression, and future studies should seek to gather student financial data from institutional databases when possible.

### 4.5. Implications for Research and Policy

Future research on FI in the college student population should seek to build upon this work to understand the differences more fully between students with marginal FS and those with all other levels of FS. Specifically, factors that contribute to lower GPAs among students that experience marginal FS should be investigated. It is unclear if the worry over food is causing poor academic performance or if other factors, such as working additional hours to prevent falling farther into FI, is detracting from academics. Prior research investigating coping skills among food insecure students found that students engage in coping behaviors, such as searching for free food events on campus or failing to purchase educational supplies, which take time and energy away from coursework or studying [42]. It is possible that students with marginal FS demonstrate similar patterns of behavior that could in part explain why they have lower GPAs than highly food secure individuals. Focus groups or interviews with marginally food secure students would allow for insight into the multiple factors that potentially cause lower grades among this demographic.

While this study fills a gap in the literature by exploring differences between students with marginal FS and all other levels of FS, it also provides policy implications that impact both student and non-student populations. The Supplemental Nutrition Assistance Program (SNAP) was recently expanded to provide increased access to benefits for college students [43], but if estimates of the number of students in need of food assistance fail to include students with marginal FS, it is likely that the program will be underfunded. Similarly, failure to include marginally FS members of the general population in national estimates of FI could lead to underfunded food programs such as SNAP, the Special Supplemental Nutrition Program for Women, Infants, and Children (WIC), and the National School Lunch Program (NSLP).

## 5. Conclusions

This study provides evidence that there are important characteristics of marginally food secure students that make them similar to food insecure students, and different from highly food secure students. When studying FI on campus, researchers should investigate and report the number of students that fall within each of the four FS levels. When a binary measure is used to describe FI in a population of students, those with high and marginal FS are currently grouped together, limiting the ability of university administrators to accurately estimate the number of students that may be in need of food or financial support. It may even be advisable to include the marginally food secure students with food insecure students, as long as it is clear how that category is calculated. University personnel should then use this information to allocate food and financial resources to students in need.

This study focuses on marginal FS in the college student population, but there are implications for the measurement of FI beyond this population, especially since measurement tools impact families, communities, and the allocation of resources. USDA measurement tools and coding guidelines are commonly used to study FI, but the current model underestimates the number of food insecure households [3]. The USDA should continue to measure FI using a four-item scale, categorizing individuals as having high, marginal, low, or very low FS. However, they should not continue to cluster highly and marginally food secure individuals together as there are noted differences between the groups [3,4,13] in both the general and college student populations. Individuals with marginal FS should be included under the label food insecure along with those with low or very low FS.

Changing this categorization will provide a more appropriate estimate of the number of people that experience any indication of FI. Coleman-Jensen [3] highlights the potential negative outcomes of the current USDA categorization stating, “A definition that underestimates prevalence rates is likely to underestimate need for food assistance, which may have implications for local, state, and federal budgets, as well as threaten the well-being of households facing food shortages” (p. 227). The COVID-19 pandemic underscored the need to appropriately estimate the number of households and college students in need of food assistance. The number and needs of marginally food secure individuals must be recognized to provide them with sufficient resources and supports.

## Figures and Tables

**Table 1:** Conditions associated with FS level according to the USDA 10-Item FS questionnaire.

	Food Security Level	USDA 10-Item Score	Associated Conditions or Behaviors
Food Secure	High FS	0	No food acquisition difficulties
	Marginal FS	1–2	Worry over sufficiency of food supply; food supply may not last
Food Insecure	Low FS	3–5	Reduced diet quality or variety; some indications of reduced food intake
	Very Low FS	6–10	Reduced food intake; disrupted eating patterns; potential weight loss

Note. FS is food security. Information in the table is from the USDA [2,9].

**Table 2:** Descriptive Statistics.

	Level of FS				
	High *n* = 1896	Marginal *n* = 747	Low *n* = 819	Very Low *n* = 1029	Total *n* = 4491
**Demographic and Student Characteristics**					
Age	20.8 (2.9)	20.8 (2.2)	20.9 (2.7)	21.2 (3.2)	20.9 (2.8)
Citizenship					
US citizen	89.9	86.6	86.2	88.0	88.3
Non-US citizen	10.1	13.4	13.8	12.0	11.7
Enrollment status					
Full-time	97.4	97.2	98.4	98.2	97.7
Part-time	2.6	2.8	1.6	1.8	2.3
First-generation college student	24.6	32.9	38.0	45.4	33.2
Race					
African American/Black	5.1	5.5	7.7	8.4	6.4
Asian	35.1	32.7	31.4	28.0	32.4
Hispanic	9.4	12.0	16.8	20.8	13.8
Other *	4.9	5.9	6.0	6.7	5.7
White	45.4	43.9	38.1	36.2	41.7
**Financing Education**					
Family pays	79.3	73.4	69.7	64.4	73.2
Federal work-study or Pell	19.0	29.0	30.0	37.9	27.0
Finance education	57.7	67.9	72.6	79.7	67.2
Friends pay	1.3	2.3	5.5	12.1	4.7
Grant, scholarship, or fellowship	39.9	41.5	36.1	41.1	39.8
Non-work-study job	31.0	31.9	34.9	37.3	33.3
**Academics**					
Cumulative GPA	3.30 (0.53)	3.19 (0.52)	3.12 (0.56)	3.01 (0.56)	3.18 (0.55)
**Other Characteristics**					
Used on-campus pantry (*n* = 958) **	0.7	1.7	3.4	3.1	1.9
Used off-campus pantry (*n* = 4431)	2.6	4.4	5.5	6.8	4.4
Has campus meal plan (*n* = 4485)	48.5	49.0	42.2	37.1	44.9
Has SNAP benefits	0.9	1.2	2.2	2.9	1.6

Note. FS is food security; GPA is grade point average; SNAP is supplemental nutrition assistance program. All percentages are out of the column n unless otherwise indicated. The mean is reported for continuous variables. * Other includes students that have an unknown race or identify as another race that does not fall within the categories White, Black or African American, Asian, or Hispanic. ** Data were only collected from students who heard of the on-campus pantry.

**Table 3:** Results of likelihood-ratio tests for the overall model.

	Chi-Square	Degrees of Freedom
**Demographic and Student Characteristics**		
Age	7.180	3
Citizenship	20.675 **	3
Enrollment status	13.848 *	3
First-Generation College Student	19.912 **	3
Race	33.960 **	12
**Financing Education**		
Family pay	45.185 **	3
Federal work-study or Pell Grant	29.652 **	3
Finance education	72.203 **	3
Friends pay	112.211 **	3
Grant, scholarship, or fellowship	12.774 *	3
Non-work-study job	10.347	3
**Academic**		
Cumulative GPA	93.633 **	3

Note. GPA is grade point average. * *p* < 0.01, ** *p* < 0.001.

**Table 4:** Results of pairwise comparisons of the multinomial logistic regression.

	High vs. Marginal			Low vs. Marginal			Very Low vs. Marginal		
Variables	b (SE)	OR	99% CI	b (SE)	OR	99% CI	b (SE)	OR	99% CI
Intercept	−1.069 (0.673)			−1.226 (0.786)			−0.907 (0.733)		
**Demographic and Student Characteristics**									
Age	0.024 (0.020)	1.024	[0.973, 1.064]	0.028 (0.022)	1.029	[0.972, 1.089]	0.051 (0.020)	1.052	[0.998, 1.108]
Citizenship	0.489 (0.144) **	1.631	[1.125, 2.365]	−0.039 (0.161)	0.962	[0.635, 1.457]	0.046 (0.160)	1.047	[0.694, 1.579]
Enrollment	0.074 (0.296)	1.078	[0.503, 2.308]	0.923 (0.388)	2.516	[0.926, 6.840]	0.961 (.360)*	2.614	[1.034, 6.609]
First-Generation Student	−0.174 (0.103)	0.841	[0.645, 1.096]	0.114 (0.116)	1.120	[0.831, 1.511]	0.231 (0.111)	1.259	[0.945, 1.678]
Race									
African American/Black	0.100 (0.202)	1.105	[0.657, 1.859]	0.456 (0.220)	1.577	[0.895, 2.778]	0.426 (0.212)	1.532	[0.888, 2.642]
Asian	0.157 (0.108)	1.169	[0.884, 1.546]	0.130 (0.130)	1.139	[0.815, 1.591]	0.091 (0.127)	1.095	[0.789, 1.520]
Hispanic	0.015 (0.152)	1.015	[0.686, 1.501]	0.424 (0.165) *	1.528	[0.999, 2.338]	0.499 (0.157) **	1.647	[1.100, 2.467]
Other	−0.151 (0.197)	0.860	[0.518, 1.428]	0.176 (0.224)	1.193	[0.670, 2.124]	0.291 (0.213)	1.338	[0.773, 2.316]
**Financing Education**									
Family pays	0.282 (0.105) *	1.326	[1.012, 1.738]	−0.154 (0.117)	0.857	[0.634, 1.159]	−0.338 (0.112) *	0.713	[0.534, 0.951]
Work-Study/Pell	−0.394 (0.118) **	0.674	[0.498, 0.913]	0.009 (0.133)	1.009	[0.716, 1.422]	0.156 (0.127)	1.169	[0.843, 1.620]
Finance education	−0.363 (0.099) **	0.695	[0.539, 0.898]	0.193 (0.120)	1.213	[0.890, 1.653]	0.409 (0.120) **	1.505	[1.104, 2.053]
Friends pay	−0.435 (0.323)	0.647	[0.282, 1.487]	0.815 (0.292) *	2.259	[1.065, 4.794]	1.586 (0.267) **	4.884	[2.453, 9.724]
Grant, scholarship, or fellowship	0.031 (0.102)	1.031	[0.792, 1.343]	−0.310 (0.121) *	0.733	[0.537, 1.002]	−0.164 (0.117)	0.849	[0.628, 1.146]
Non-work-study job	−0.037 (0.098)	0.964	[0.749, 1.241]	0.165 (0.113)	1.179	[0.882, 1.576]	0.227 (0.108)	1.254	[0.949, 1.658]
**Academic**									
GPA	0.344 (0.087) **	1.410	[1.126, 1.765]	−0.100 (0.098)	0.905	[0.703, 1.164]	−0.414 (0.093) **	0.661	[0.520, 0.841]

Note. GPA is grade point average. The number of students in each category is *n* = 1896 for high FS, *n* = 747 for marginal FS, *n* = 879 for low FS, and *n* = 1029 for very low FS. * *p* < 0.01, ** *p* < 0.001. R^2^ = 0.158 (Nagelkerke). Model x^2^ (45) = 710.00, *p* < 0.001.

## Data Availability

The data presented in this study are available on request from the corresponding author. The data are not publicly available due to privacy of the research participants.

## Supplementary Materials

The following supporting information can be downloaded at: https://www.mdpi.com/article/10.3390/nu14153142/s1, File S1: Survey Questions Used to Describe the Sample and Used in Multinomial Logistic Regression; Table S1: Sample Representativeness of Undergraduate Student Population.
