# Supplementary material for: Underestimating College Student Food Insecurity: Marginally Food Secure Students May Not Be Food Secure
Source: Nutrients. 2022 Jul 29;14(15):3142. doi: 10.3390/nu14153142 (PMC9370637; doi:10.3390/nu14153142)
Supplement: Supplementary file 1 [file nutrients-14-03142-s001.zip › nutrients-1804563-Supp File S1.pdf]

Survey Questions Used to Describe the Sample[1] and Used in Multinomial Logistic Regression

LEVEL OF FOOD SECURITY (USDA 10-Item Food Security Survey Module):

Thinking about the last 30 days, how true would you say the following statements are?

I was worried whether my food would run out before I had money to buy more.

- ❍ **Often True (1)**
- ❍ **Sometimes True (2)**
- ❍ **Never True (3)**
- ❍ **I don't know (4)**

The food that I bought just didn’t last, and I didn’t have money to get more.

- ❍ **Often True (1)**
- ❍ **Sometimes True (2)**
- ❍ **Never True (3)**
- ❍ **I don't know (4)**

I couldn't afford to eat balanced meals.

- ❍ **Often True (1)**
- ❍ **Sometimes True (2)**
- ❍ **Never True (3)**
- ❍ **I don't know (4)**

Display This Question:

If I was worried whether my food would run out before I had money to buy more. Never True Is Not Selected

Or The food that I bought just didn’t last, and I didn’t have money to get more. Never True Is Not Selected

---

[1] Other items used to describe the student sample including age, citizenship, enrollment status, first generation college student, race, and cumulative GPA were provided by the intuition’s Office of Institutional Research.

Or I couldn't afford to eat balanced meals. Never True Is Not Selected

In the last 30 days, did you ever cut the size of your meals or skip meals because there wasn't enough money for food?

- ❍ **Yes (1)**
- ❍ **No (2)**
- ❍ **I don't know (3)**

Display This Question:

If In the last 30 days, did you ever cut the size of your meals or skip meals because there wasn't enough money for food? Yes Is Selected

How many of the last 30 days did you cut the size of your meals or skip meals because there wasn't enough money for food? [OPEN]

Display This Question:

If I was worried whether my food would run out before I had money to buy more. Never True Is Not Selected

Or The food that I bought just didn’t last, and I didn’t have money to get more. Never True Is Not Selected

Or I couldn't afford to eat balanced meals. Never True Is Not Selected

In the last 30 days, did you ever eat less than you felt you should because there wasn’t enough money for food?

- ❍ **Yes (1)**
- ❍ **No (2)**
- ❍ **I don't know (3)**

Display This Question:

If I was worried whether my food would run out before I had money to buy more. Never True Is Not Selected

Or The food that I bought just didn’t last, and I didn’t have money to get more. Never True Is Not Selected

Or I couldn't afford to eat balanced meals. Never True Is Not Selected

In the last 30 days, were you ever hungry but didn't eat because there wasn't enough money for food?

- **Yes (1)**
- **No (2)**
- **I don't know (3)**

Display This Question:

If I was worried whether my food would run out before I had money to buy more. Never True Is Not Selected

Or I couldn't afford to eat balanced meals. Never True Is Not Selected

Or The food that I bought just didn't last, and I didn't have money to get more. Never True Is Not Selected

In the last 30 days, did you lose weight because there wasn't enough money for food?

- **Yes (1)**
- **No (2)**
- **I don't know (3)**

Display This Question:

If I was worried whether my food would run out before I had money to buy more. Never True Is Not Selected

Or The food that I bought just didn't last, and I didn't have money to get more. Never True Is Not Selected

Or I couldn't afford to eat balanced meals. Never True Is Not Selected

In the last 30 days, did you ever not eat for a whole day because there wasn't enough money for food?

- **Yes (1)**
- **No (2)**
- **I don't know (3)**

Display This Question:

If In the last 30 days, did you ever not eat for a whole day because there wasn't enough money for food? Yes Is Selected

For how many of the last 30 days did you not eat for a whole day because there wasn't enough money for food? [OPEN ENDED]

MECHANISMS OF FINANCING EDUCATION

In which of the following ways did you pay for the expenses associated with attending college this semester? This includes tuition, fees and living expenses. Please check all that apply.

- ❑ **I get help from my family**
- ❑ **I take out student loans**
- ❑ **I have a scholarship**
- ❑ **I have a Teaching Assistantship (TA) or Graduate Assistantship (GA)**
- ❑ **I have a work-study job**
- ❑ **I have a job that isn't a work study job, TA or GA**
- ❑ **I have a fellowship**
- ❑ **I get a Pell Grant**
- ❑ **I get a grant other than a Pell grant from the federal or state government**
- ❑ **I get a grant from a government other than the US government**
- ❑ **I get a grant from (institution name)**
- ❑ **I use my savings**
- ❑ **I use my credit cards**
- ❑ **I get help from friends**
- ❑ **My employer pays**
- ❑ **Other, please explain ____________________**

Survey Questions Used to Describe the Sample that Were Not Included in the Multinomial Logistic Regression

Have you heard about the (institution name) Food Pantry, a pantry that provides food for students in need?

- ❍ **No (1)**
- ❍ **Yes (2)**
- ❍ **I am not sure (3)**

Display This Question:

If Have you heard about the (institution name) Food Pantry, a pantry that provides food for stu... Yes Is Selected

During the current semester, have you received food from the (institution name) Food Pantry?

- ❍ **Yes (1)**
- ❍ **No (2)**

During the current semester, have you received food from an off-campus food pantry?

- ❍ **Yes (1)**
- ❍ **No (2)**

Do you have a (institution name) meal plan for Fall 2016 semester?

- ❍ **Yes (1)**
- ❍ **No (2)**

Please indicate any federal, state, or local assistance you may have received over the past 12 months. Check all that apply.

- ❑ **I do not receive any kind of federal, state or local assistance. (1)**
- ❑ **SNAP (food stamps) (2)**
- ❑ **WIC (nutritional assistance for pregnant women and children) (3)**
- ❑ **TANF (public cash assistance; formerly called ADC or ADFC) (4)**
- ❑ **SSI (supplemental security income) (5)**

- ❑ **SSDI (social security disability income) (6)**
- ❑ **Medicaid or Public health insurance (7)**
- ❑ **Child care assistance (8)**
- ❑ **Unemployment compensation/insurance (9)**
- ❑ **Utility assistance (10)**
- ❑ **Housing assistance (11)**
- ❑ **Transportation assistance (12)**
- ❑ **Tax rebates or tax credits (13)**
- ❑ **Veterans benefits (Veteran's Administration benefits for a servicemen's, widow's, or survivor's pension, service disability or the GI bill (14)**
