# Supplementary material for: Underestimating College Student Food Insecurity: Marginally Food Secure Students May Not Be Food Secure
Source: Nutrients. 2022 Jul 29;14(15):3142. doi: 10.3390/nu14153142 (PMC9370637; doi:10.3390/nu14153142)
Supplement: Supplementary file 1 [file nutrients-14-03142-s001.zip › nutrients-1804563-supplementary.pdf]

Table S1. Sample Representativeness of Undergraduate Student Population.

	Total N	Percent	Response N	Response Rate	Change	Expected Sample Value	Difference (Expected - Observed)	Difference^ 2/ Expected	Chi-Square Value	P-Value	Cramers V
**TOTAL**	34,596		6,823	19.72%							
**Citizenship**									19.80	0.00	0.02
US Citizen	29,447	85.12%	5,939	20.17%	0.45%	5808	-131	2.98			
Permanent Resident	2,176	6.29%	401	18.43%	-1.29%	429	28	1.85			
Foreign National	2,920	8.44%	483	16.54%	-3.18%	576	93	14.98			
**Class Level: Total**									104.18	0.00	0.05
First Year	7,124	20.59%	1,702	23.89%	4.17%	1405	-297	62.79			
Sophomore	7,817	22.60%	1,566	20.03%	0.31%	1542	-24	0.38			
Junior	8,778	25.37%	1,705	19.42%	-0.30%	1731	26	0.40			
Senior	10,877	31.44%	1,850	17.01%	-2.71%	2145	295	40.61			
**Gender**									685.32	0.00	0.14
Female	16,976	49.07%	4,429	26.09%	6.37%	3348	-1081	349.04			
Male	17,620	50.93%	2,394	13.59%	-6.14%	3475	1081	336.28			
**School**									199.01	0.00	0.08
School A	183	0.53%	53	28.96%	9.24%	36	-17	7.92			
School B	863	2.49%	228	26.42%	6.70%	170	-58	19.63			
School C	788	2.28%	123	15.61%	-4.11%	155	32	6.76			
School D	3,776	10.91%	700	18.54%	-1.18%	745	45	2.68			
School E	264	0.76%	67	25.38%	5.66%	52	-15	4.28			
School F	21,043	60.82%	3,910	18.58%	-1.14%	4150	240	13.89			
School G	3,857	11.15%	732	18.98%	-0.74%	761	29	1.08			
School H	3,422	9.89%	967	28.26%	8.54%	675	-292	126.44			
School I	400	1.16%	43	10.75%	-8.97%	79	36	16.33			
**Full/ Part Time**									83.54	0.00	0.05
Full Time	33,083	95.63%	6,679	20.19%	0.47%	6525	-154	3.65			
Part Time	1,513	4.37%	144	9.52%	-10.20%	298	154	79.89			
**Race/Ethnicity**									3.75	0.44	0.01
African American	2,559	7.40%	515	20.13%	0.40%	505	-10	0.21			
Asian	11,707	33.84%	2259	19.30%	-0.43%	2309	50	1.08			
Hispanic	4,577	13.23%	944	20.62%	0.90%	903	-41	1.89			
White	13,984	40.42%	2743	19.62%	-0.11%	2758	15	0.08			
Other	1,769	5.11%	362	20.46%	0.74%	349	-13	0.49			
**Financial Aid**											
Pell	11,946	34.53%	2,571	21.52%	1.80%	2356	-215	19.62	29.97	0.00	0.03
No Pell	22,650	65.47%	4,252	18.77%	-0.95%	4467	215	10.35			
Any Financial Aid	26,182	75.68%	5,493	20.98%	1.26%	5164	-329	21.01	86.40	0.00	0.05
No Financial Aid	8,414	24.32%	1,330	15.81%	-3.91%	1659	329	65.39			

Note. Individual schools within the University are labeled alphabetically to protect the identity of the institution. The race category 'Other' includes American Indian, Other, Two or More and Unknown. Federal Pell Grant data reported in this table was provided by the Office of Institutional Research, but this data was not available at the individual student level. Self-report data on Pell Grant status was also collected and used in analyses.
